# Altered *Ex-Vivo* Cytokine Responses in Children With Asymptomatic *Plasmodium falciparum* Infection in Burkina Faso: An Additional Argument to Treat Asymptomatic Malaria?

**DOI:** 10.3389/fimmu.2021.614817

**Published:** 2021-06-09

**Authors:** Annelies Post, Berenger Kaboré, Mike Berendsen, Salou Diallo, Ousmane Traore, Rob J. W. Arts, Mihai G. Netea, Leo A. B. Joosten, Halidou Tinto, Jan Jacobs, Quirijn de Mast, André van der Ven

**Affiliations:** ^1^ Department of Internal Medicine, Radboud Centre for Infectious Diseases, Radboud University Medical Centre, Nijmegen, Netherlands; ^2^ IRSS/Clinical Research Unit of Nanoro (CRUN), Nanoro, Burkina Faso; ^3^ Bandim Health Project, Institute of Clinical Research, University of Southern Denmark and Odense University Hospital, Odense, Denmark; ^4^ Department for Genomics & Immunoregulation, Life and Medical Sciences Institute (LIMES), University of Bonn, Bonn, Germany; ^5^ Institut Supérieur des Sciences de la Santé, Université Nazi Boni de Bobo-Dioulasso, Bobo-Dioulasso, Burkina Faso; ^6^ Department of Clinical Sciences, Institute of Tropical Medicine, Antwerp, Belgium; ^7^ Department of Microbiology, Immunology and Transplantation, KU Leuven, Leuven, Belgium

**Keywords:** asymptomatic malaria, bacteraemia, Salmonella, iNTS, bloodstream infection

## Abstract

**Introduction:**

Patients with clinical malaria have an increased risk for bacterial bloodstream infections. We hypothesized that asymptomatic malaria parasitemia increases susceptibility for bacterial infections through an effect on the innate immune system. We measured circulating cytokine levels and *ex-vivo* cytokine production capacity in asymptomatic malaria and compared with controls.

**Methods:**

Data were collected from asymptomatic participants <5 years old with and without positive malaria microscopy, as well as from hospitalized patients <5 years old with clinical malaria, bacteremia, or malaria/bacteremia co-infections in a malaria endemic region of Burkina Faso. Circulating cytokines (TNF-α, IFN-γ, IL-6, IL-10) were measured using multiplex assays. Whole blood from asymptomatic participants with and without positive malaria microscopy were *ex-vivo* stimulated with *S. aureus*, *E. coli* LPS and *Salmonella* Typhimurium; cytokine concentrations (TNF-α, IFN-γ, IL-1β, IL-6, IL-10) were measured on supernatants using ELISA.

**Results:**

Included were children with clinical malaria (n=118), bacteremia (n=22), malaria and bacteremia co-infection (n=9), asymptomatic malaria (n=125), and asymptomatic controls (n=237). Children with either clinical or asymptomatic malaria had higher plasma cytokine concentrations than controls. Cytokine concentrations correlated positively with malaria parasite density with the strongest correlation for IL-10 in both asymptomatic (r=0.63) and clinical malaria (r=0.53). Patients with bacteremia had lower circulating IL-10, TNF-α and IFN-γ and higher IL-6 concentrations, compared to clinical malaria. *Ex-vivo* whole blood cytokine production to LPS and *S. aureus* was significantly lower in asymptomatic malaria compared to controls. Whole blood IFN-γ and IL-10 production in response to *Salmonella* was also lower in asymptomatic malaria.

**Interpretation:**

In children with asymptomatic malaria, cytokine responses upon *ex-vivo* bacterial stimulation are downregulated. Further studies are needed to explore if the suggested impaired innate immune response to bacterial pathogens also translates into impaired control of pathogens such as *Salmonella spp*.

## Introduction

*Plasmodium falciparum* infections are a risk factor for bacterial bloodstream infections (bBSI) (1, 2), especially those caused by gram negative bacteria. Non-typhoidal *Salmonellae* are among the most commonly found isolates in blood cultures among children in sub-Saharan Africa (SSA) (3–5), both among patients with and without co-occurring malaria infection. The case fatality rate of invasive non-Typhoidal *Salmonella (*iNTS) bloodstream infection is estimated at 20% (6). Patients with a co-infection malaria and iNTS have an increased risk of mortality compared to patients with only malaria or iNTS infection.

Pediatric iNTS and malaria overlap in terms of geographical distribution, age distribution, and seasonality. Several studies showed that a decreased malaria incidence in a particular region was associated with a decreased iNTS incidence (7, 8). Different mechanisms may underlie the epidemiologic association between malaria and iNTS infections (9). First, the intestinal mucosal barrier function is disturbed during malaria, facilitating invasion and subsequently infection. Second, malaria is reported to cause temporary neutrophil (10) and macrophage dysfunction, and influences the cytokine production capacity of immune cells which could alter susceptibility to iNTS (11). Third, changes in iron homeostasis with increased storage of iron within macrophages contributes to a suitable growing environment for *Salmonella* infection (12).

So far, studies investigating the association between bBSI and malaria are mostly limited to clinical malaria and animal models. In many malaria-endemic areas, asymptomatic malaria parasitemia is highly prevalent, but it is unknown whether asymptomatic malaria increases the risk for bBSI by changes in the host immune responses (9, 13). In the present study, we analyzed circulating cytokine concentrations in asymptomatic healthy children below the age of five with or without parasitemia and children with acute febrile illness diagnosed with malaria, bBSI or co-infections. Furthermore, we compared cytokine production capacity in asymptomatic healthy children with or without parasitemia as an indicator of the presence of immune tolerance.

## Methods

### Study Site and Study Objectives

Samples were obtained from two studies conducted between March 2016 and September 2017 at the Clinical Research Unit of Nanoro (CRUN), a rural research facility situated in the central-west region of Burkina Faso, at approximately 85km from the capital city Ouagadougou (14). With an estimated 7.9 million cases per year in 2017, Burkina Faso has one of the highest incidences of malaria in SSA (15). Peak incidences coincide with the rainy season (July-October), but cases occur year round. Most cases are caused by *Plasmodium falciparum*, but *Plasmodium ovale* and *Plasmodium malariae* are also sporadically found (3, 16). Seasonal Malaria Chemoprophylaxis (SMC) for children under 5 years was introduced in 2016.

The objective of this study was to assess the effect of (asymptomatic) malaria on cytokine responses to bacterial pathogens among children below the age of five, since children of this age group are most likely to suffer from severe malaria and iNTS infection. We hypothesized that patients with clinical malaria and asymptomatic malaria have a lower cytokine response to bacterial pathogens compared to patients who are malaria microscopy slide negative. To assess our hypothesis, we aggregated data from two studies simultaneously being carried out at CRUN on respectively a healthy population (ClinicalTrials.gov identifier: NCT03176719) and a population presenting with acute febrile illness (AFI) (ClinicalTrials.gov identifier: NCT02669823). For the current study, all participants from either study who were less than 5 years old, were selected.

The first study was an explorative cross-sectional study among healthy volunteers of 1 year and older living in the Health and Demographic Surveillance System (HDSS) area of Nanoro. The HDSS area of Nanoro covers a population of approximately 65.000 inhabitants distributed over 24 villages. Study methods are described in detail elsewhere (Kaboré, submitted; ClinicalTrials.gov identifier: NCT03176719). Briefly, healthy participants of 1 year and older were randomly selected from the 24 villages of the HDSS catchment area. Informed consent was obtained from their parents or legal guardians. After inclusion, an extensive electronic questionnaire concerning health and demographic data was obtained. Finally, EDTA anti-coagulated and heparinized blood was collected and transported back to CRUN in a cool box for hemocytometry, malaria diagnostics and *ex-vivo* whole blood stimulation. Supernatants were stored at -80°C and shipped to the Netherlands for cytokine analyses.

The second study was a phase-three diagnostic accuracy study to assess the diagnostic performance of two novel technologies to differentiate malaria, bacterial infections, and viral infections. Study methods are described in detail elsewhere (17). In summary, participants of three months and older presenting with AFI at the referral hospital of Nanoro were asked to participate in the study. After consent was obtained basic clinical and demographic data were registered. After inclusion, EDTA anti-coagulated blood and an aerobic blood culture were obtained from each patient and transported to the laboratory. Malaria diagnostics and blood culture were performed on site. One milliliter whole-blood and plasma samples were stored at -80°C and shipped to the Netherlands for retrospective malaria- and bacterial PCRs, as well as assessment of circulating cytokines.

### Laboratory Procedures

Upon arrival at the clinical research laboratory of CRUN, the EDTA blood samples were analyzed using an XN-1000 automated hematology analyzer (Sysmex Corporation, Kobe, Japan). Complete blood counts (CBC) and leukocyte differential were recorded.

### Malaria Diagnostics

Thick and thin blood films were made using 10μL EDTA-anticoagulated blood. Slides were stained with 3% Giemsa solution and examined for presence of *Plasmodium* parasites according to the World Health Organization (WHO) procedures (18). Results were expressed as asexual parasites/µl using the White Blood Cell (WBC) count as measured by hematology analyzer. At least 200 white blood cells were counted. Slides were viewed by two independent microscopists. In case of discrepancies between the readers’ results (*e.g.* discrepancy between positive and negative slides, a more than 1 log difference in parasite density, or discrepancy in *Plasmodium* species) the results of a third blinded microscopist was decisive. Malaria diagnostics and hematology analysis were done within 6 hours after sample collection.

### *Ex-Vivo* Whole Blood Stimulations

A heparinized whole blood sample was collected from participants in the cross-sectional study for *ex*-*vivo* whole blood stimulations. The whole blood stimulations were done at the research laboratory of CRUN within 12 hours after blood collection. Three stimuli (10ng/ml) *Escherichia coli* LPS (serotype 055:B5, Sigma-Aldrich), 1x10ˆ6/ml heat killed *Staphylococcus aureus* (clinical isolate) and 1x10ˆ6/ml heat-killed *Salmonella* Typhimurium (ST-313 wildtype, obtained from blood culture in the Democratic Republic of the Congo) and a negative control medium (RPMI-1640 Dutch modification (Life Technologies, Carlsbad, California, USA) supplemented with gentamicin 50μg/ml, glutamax 2mM, pyruvate 1mM) were used for each participant.

Blood was diluted at 1/5 in RPMI and distributed over four wells per participant in a final concentration of 500 µl. Each well was stimulated with one of four stimuli at an end concentration of 1x10^6^/ml and incubated at 37°C. After 48 hours, plates were centrifuged at 400g for 5 minutes. Supernatant aliquots of 300 µl were collected and stored in Micronics tubes (Lelystad, The Netherlands) at -80°C. After completion of the trial, all supernatants were transported to Radboudumc Nijmegen on dry ice for batch analysis of cytokine levels. Participants with an IL-1β and TNF-α response in the RPMI control sample were considered contaminated and were therefore excluded from analyses.

### Cytokine Measurements in Supernatants After Whole Blood Stimulation

Cytokines were assessed using commercial ELISA kits for human TNF-α, IFN-γ, IL-1β, IL-10 (R&D systems, MN, USA) and human IL-6 (Sanquin, Amsterdam, the Netherlands) according to manufacturer’s instructions. All assays were performed in a timespan of two consecutive days: RPMI and *Staphylococcus aureus* were measured on day one, *Salmonella* Typhimurium and *Escherichia coli* LPS were measured on day two. Samples were allowed to thaw at 4°C one day prior to analysis. Plate set-up was randomized in terms of participant age, presence of asymptomatic malaria and month of storage to decrease batch-effect. Reported cytokine concentrations are based off standard curves which were ran on each plate.

### Circulating Cytokines Measurements

Circulating cytokines of patients participating to both studies were batch-tested, using stored EDTA plasma samples. Plate set-up was randomized as previously described. The pro-inflammatory cytokines IL-6, TNF-α, IFN-γ and anti-inflammatory cytokine IL-10 were quantified using the MAGPIX technology (Luminex Corporation, Austin, Texas, USA) with the Human High Sensitivity Cytokine Premixed Magnetic Luminex Performance Assay kits from Bio-Techne (R&D systems). Manufacturer’s recommendations were followed during the assay.

### Case Definitions

Asymptomatic malaria was defined as one or more parasites in thick smear malaria microscopy without fever or other acute symptoms. Clinical malaria was defined as febrile patients with one or more malaria parasites in thick smear microscopy and without other possible explanations for the fever. Bacteremia was defined as a blood culture grown with a pathogenic bacterium. Combined malaria and bacteremia was recorded in case of simultaneous blood culture grown with a pathogenic bacterium and one or more malaria parasites in malaria thick smear microscopy. Patients with a combined malaria and bacteremia are hereafter referred to as “co-infection” or “*Salmonella* co-infection” in case of combined malaria and *Salmonella*. Anemia was defined by age and sex according to WHO classification. Malnutrition was recorded as patients with a low weight-for-height (wasting) or height-for-age (stunting) according to WHO guidelines.

### Statistical Analyses

As appropriate, means or medians were compared using a two-sample t-test, Mann-Whitney U test or Kruskal-Wallis. Differences in *ex-vivo* stimulated cytokine production between participants with and without asymptomatic malaria were corrected for using a multivariable regression, multicollinearity was assessed using a correlation matrix. Data were log-transformed for analysis in case of non-normal distribution. For normally distributed cytokine measurements a linear regression model was used. A Tobit regression was used for analyses with results outside of the upper or lower quantification limits. The association between cytokine production and parasite density was measured using a Pearson’s correlation or Spearman’s correlation as appropriate. Graphs and figures were created using Graphpad (Graphpad Prism, version 7.00, La Jolla, California, USA). A p-value of.05 was considered statistically significant. Results were corrected for multiple testing using a Bonferroni correction.

### Ethical Considerations

Both study protocols were approved by the national health ethics committee of Burkina Faso (ref 2016-01-006 and 2015-01-006 respectively). The diagnostic accuracy study was furthermore approved by the internal review board of IRSS (ref A03-2016/CEIRES) the ethical committee of the Antwerp University Hospital (ref 15/47/492) and the institutional review board of the Institute of Tropical Medicine Antwerp (ref 1029/15).

## Results

### Baseline Characteristics

**Figure 1:**
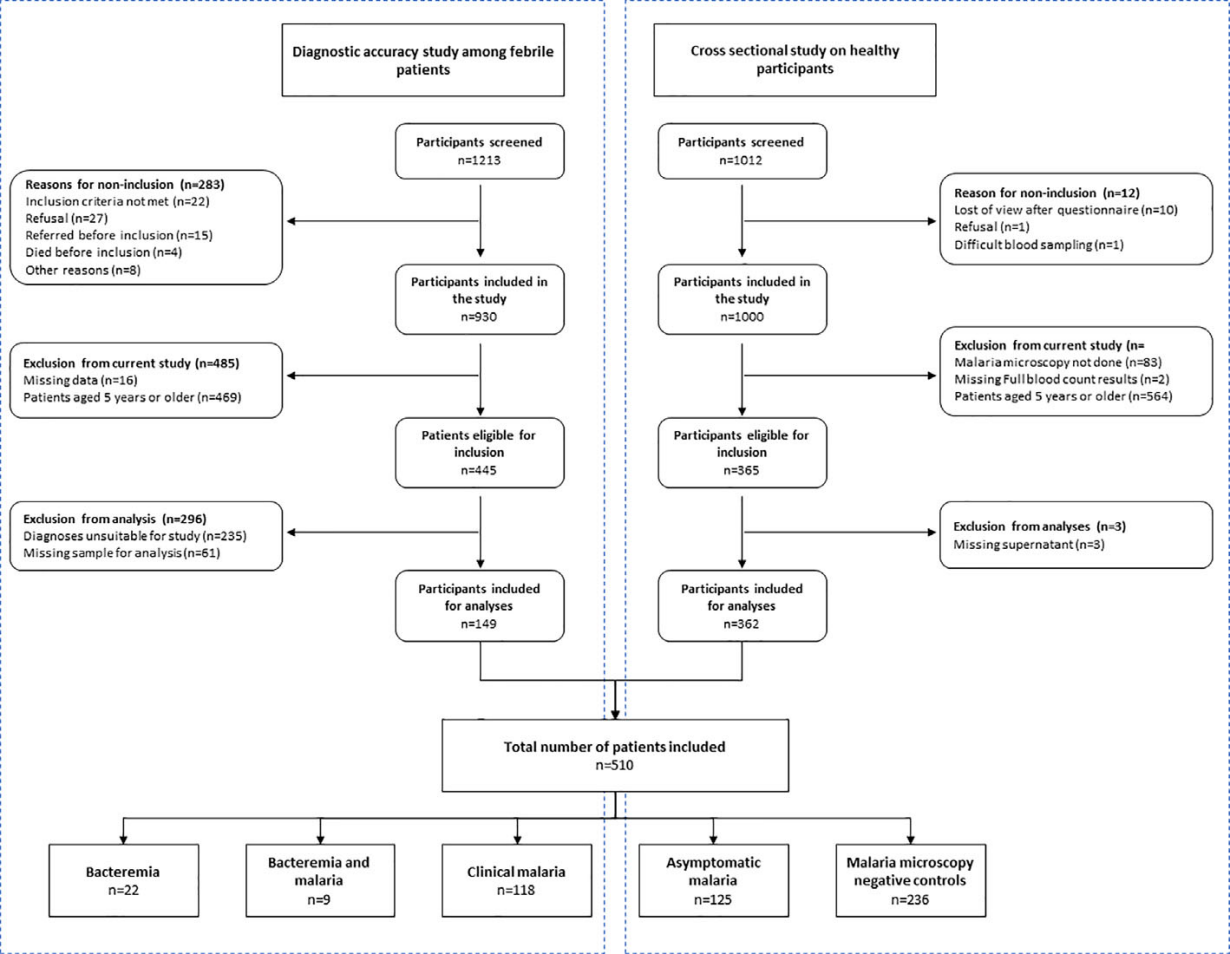
Baseline characteristics of participants included in the current study; derived from participants to the diagnostic accuracy study among febrile patients and the cross-sectional study on healthy participants.

A total of 510 participants were included in this study: 361 from the cross-sectional study among healthy participants, and 149 from the clinical diagnostic accuracy study (**Figure 1**).

The cross-sectional study enrolled 1000 participants between June and October 2016, of whom 449 (44.6%) had asymptomatic malaria. Out of 399 eligible participants, a total of 361 (90.5%) participants were included in analysis; reasons for exclusion were missing malaria microscopy data (n=33), missing demographic data (n=2) and missing supernatant samples (n=3). Among included participants, 125 (34.6%) had asymptomatic malaria.

The diagnostic accuracy study enrolled 930 patients between March 2015 and July 2016, of whom 461 (49.6%) were between three months and five years old. In total 171 of them were eligible for inclusion in the current study: 171 patients had clinical malaria, 30 patients had bacteremia (*Salmonella* spp n=18, streptococci n=5, *Escherichia coli* n=3, *Haemophilus influenzae* n=2, *Neisseria meningitidis* n=1 and one double infection) and 9 patients had a co-infection (*Salmonella* spp. n=6, and one of each *Streptococcus pneumoniae, Neisseria meningitidis* and *Acinetobacter baumannii*). Residual blood was available from 149/171 (87.1%) patients; n=118 (69.0%) with clinical malaria, n=22 (73.3%) with bacteremia and n=9 (69.2%) with a co-infection.

Baseline characteristics are presented in **Table 1**. Among participants from the cross-sectional study, baseline characteristics between participants with asymptomatic malaria and slide negative participants were mostly similar. The number of males was slightly higher among participants with asymptomatic malaria, and participants with asymptomatic malaria had a significantly higher monocyte count (1.1x10^3^ cells *versus* 0.9x10^3^ cells; p<.0001, lower Hb (10.3 g/dL *versus* 9.5 g/dL; p<.0001), Reticulocyte count (7.6 *versus* 10.8; p<.0001) and lower platelet count (382 x10^3^ cells versus 263 x10^3^ cells; p<.0001) compared to slide negative participants, but we found no significant difference in age, height, weight, middle upper arm circumference (MUAC), axillary temperature, white blood cell count or neutrophil count between both groups.

**Table 1:** Baseline characteristics.

	Cross sectional study			Diagnostic accuracy study			
	Slide negative participants	Asymptomatic Malaria		Patients with clinical malaria	Patients with Bacteremia*	Combined malaria and bacteremia**	
	n=236	n=125	*p-value*	n=114	n=22	n=9	*p-value*
Male sex (%)	116 (49.4%)	63 (50.0%)		69 (60.5%)	11 (50.0%)	4 (44.4%)	
Age (months)	25.0 (21.9–41.1)	26.4 (23.3–39.6)	.3	23.4 (13.7-36.5)	16.2 (11.7-26.7)	25.3 (23.6-36.9)	.09
Weight (kg)	11.2 (10.0–12.6)	11.4 (10.0–12.9)	.3	9.4 (8.1-11.3)	8.2 (7.0-10.2)	10.6 (9.2-11.2)	.09
Height (cm)	83 (78–90.5)	83 (78–92)	.9	81 (74-90)	77 (70-85)	82 (80-91)	.3
Height for age (Z-score)	-1.9 (-2.7/-1.2)	-2.1 (-3.2/-1.2)	.9	-1.3 (-2.3/-0.4)	-1.4 (-1.9/0.4)	-1.8 (-2.4/-1.3)	.4
MUAC (cm)	15.2 (1.2)	15.3 (1.1)	.2	NA	NA	NA	
Axillary temperature (°C)	36.5 (36.1-37.1)	36.5 (36.1-36.9)	.8	38.7 (38.0-39.8)	38.5 (38.0-39.4)	39.0 (38.8-39.8)	.4
Days of fever	NA	NA		2.4 (1.0)	2.8 (1.4)	1.9 (0.6)	.09
Pulse (/min)	NA	NA		125 (118-136)	130 (109-141)	114 (100-146)	.8
Systolic pressure (mm/Hg)	NA	NA		97 (87-105)	95 (86-101)	98 (96-118)	.2
Diastolic pressure (mm/Hg)	NA	NA		61 (53-65)	61 (51-63)	62 (56-62)	.9
Breathing frequency (/min)	NA	NA		34 (32-41)	38 (32-42)	35 (33-36)	.3
Impaired consciousness	NA	NA		17 (14.4%)	1 (4.6%)	1 (11.1%)	.4
Hemoglobin (g/dl)	10.3 (1.4)	9.5 (1.7)	**<.0001**	7.2 (2.5)	7.2 (2.3)	6.3 (1.9)	.5
Reticulocytes	7.6 (5.5)	10.8 (7.2)	**<.0001**	11.2 (8.0)	5.4 (4.1)	7.9 (3.6)	**.0009**
Platelets (10^3^/µl)	382 (159)	263 (144)	**<.0001**	173 (108)	256 (199)	153 (97)	.1
Leukocyte count (10^3^/µl)	9.9 (3.2)	10.2 (3.1)	.07	11.6 (9.1-18.2)	11.9 (10.4-15.2)	9.3 (5.8-20.2)	.6
Monocyte count (10^3^/µl)	0.9 (0.4)	1.1 (0.5)	***<.0001***	1.1 (0.6-1.9)	0.9 (0.7-1.4)	0.7 (0.5-2.1)	.6
Neutrophil count (10^3^/µl)	3.0 (1.65)	2.8 (1.9)	.07	6.0 (4.2-7.9)	5.5 (3.5-8.4)	6.1 (2.2-9.0)	.8
Lymphocyte count (10^3^/µl)	5.4 (2.0)	4.9 (2.2)	.2	4.5 (2.6-6.9)	5.3 (3.6-6.9)	3.9 (1.7-9.0)	.6

MUAC, Mid-upper arm circumference | * Salmonella (n=13), Escherichia coli (n=2), Haemophilus influenzae (n=2), Streptococci (n=4), Neisseria meningitidis (n=1) | ** Salmonella spp (n=6), Acinetobacter baumannii (n=1), Streptococcus pneumoniae (n=1), Neisseria meningitidis (n=1) | *** Calculated using chi2 |.

Data is presented as mean (SD) in case of normally distributed data and median (25-75 IQR) in case of not normally distributed data. Significance value calculated using chi-2 for binary values, Mann-Whitney U-test in case of not normally distributed and student’s t-test in case of normally distributed values. A Kruskal-Wallis test was done for continuous dependent variables in the diagnostic accuracy study. Parameters with a p-value of 0.1 or less were considered potential confounders.

The bold values refer to statistically significant differences.

Among participants in the diagnostic accuracy study, differences between patients with clinical malaria, bacteremia and co-infection were more pronounced, though this was also more difficult to interpret due the variability in numbers of cases. Patients with bacteremia were in median less than 1.5 years old, whereas patients with malaria and those with co-infection were approximately 2 years old. The height-for-age among patients with co-infection was considerably lower compared to patients with malaria and patients with bacteremia, suggesting a correlation with chronic malnutrition. Patients with co-infection typically presented at the hospital earlier (1.9 day after onset of fever) compared to patients with clinical malaria (mean 2.4 days) and bacteremia (mean 2.8 days) and had a slightly higher temperature at presentation. As expected, patients from the diagnostic accuracy study had higher neutrophil counts compared to participants to the cross-sectional study. The median numbers of white blood cells were comparable between both groups.

### Circulating Cytokines and Correlation to Parasite Density

**Figure 2:**
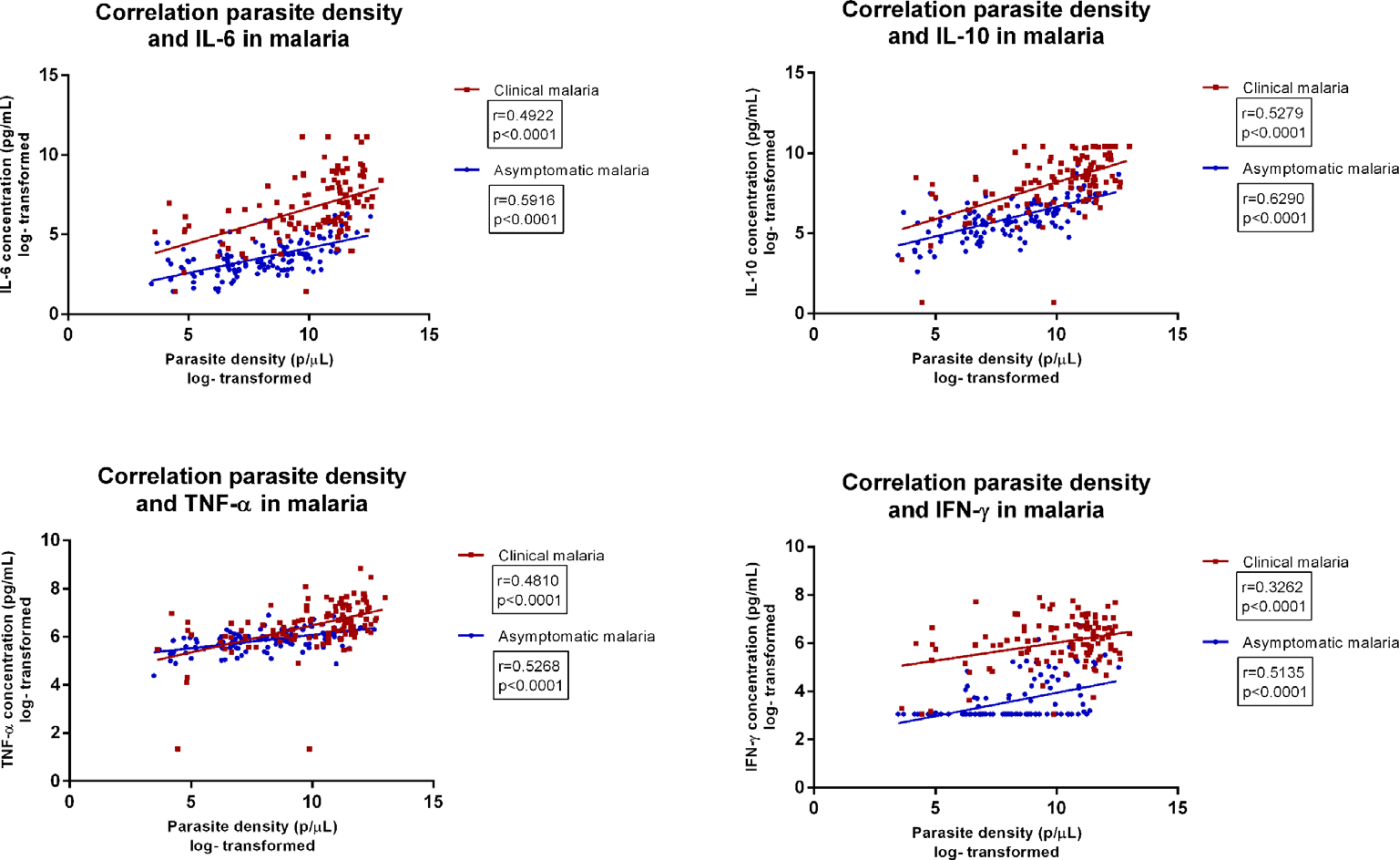
Correlation between parasite density and concentrations of various circulating cytokines in asymptomatic malaria and clinical malaria, among patients below the age of five. Legend | parasite densities reported in parasites/µL. Cytokine concentrations are reported in pg/mL.

We first assessed circulating cytokine (IL-6, IL-10, TNF-α, IFN-3γ) concentrations among healthy participants with- and without asymptomatic malaria and patients with clinical malaria. All cytokines were highest among patients with clinical malaria (**Table 2**) (19, 20) Participants with asymptomatic malaria also had significantly higher circulating cytokines compared to healthy participants without asymptomatic malaria. The median parasite density among patients with clinical malaria was 50,882 parasites/µL (IQR 7,587-104,497) compared to 3,517 parasites/µL (IQR 745-19,282) among participants with asymptomatic malaria (p<.0001). Circulating cytokine concentrations and parasite density significantly correlated for both asymptomatic and clinical malaria (**Figure 2**). The correlation was strongest for IL 10 (r=0.63 p>.0001 and r=0.53, p<.0001 respectively), followed by IL6 (r=0.59, p<.0001) in asymptomatic malaria, and TNF-α in clinical malaria (r=0.48, p<0.001). The ratio between IL-10 levels and parasite density was 0.008 and 0.01 for clinical and asymptomatic malaria respectively, whereas for IL-6 the ratio was 0.0014 *versus* 0.00088, and for TNF-α 0.0014 *versus* 0.010.

**Table 2:** Circulating cytokine concentrations among malaria slide negative participants, participants with asymptomatic malaria and patient with clinical malaria.

	Slide negative participants	Asymptomatic malaria	Patients withclinical malaria	p-value	Dunn’s test		
	n=235	n=126	n=118		SN *versus* AM	SN *versus* CM	AM versus CM
IL-6	1.2 (0.7-2.4)	3.4 (2.1-12.2)	69.2 (22.2-303.0)	<.0001	<.0001	<.0001	<.0001
IL-10	3.8 (2.4-7.7)	56.2 (29.3-132.0)	439.0 (140.0-1,333.0)	<.0001	<.0001	<.0001	<.0001
TNF-a	25.28 (20.2-30.6)	39.57 (30.6-53.5)	71.6 (42.5-121.0)	<.0001	<.0001	<.0001	<.0001
IFN-y	2.1 (2.1-2.1)	2.1 (2.1-6.8)	42.9 (25.1-78.1)	<.0001	.006	<.0001	<.0001

SN, slide negative; AM, asymptomatic malaria; CM, clinical malaria.

Data is presented as median (25-75 IQR). Significance value calculated using a Kruskal Wallis test with post-hoc Dunn’s test. Cytokine concentrations are reported in pg/mL.

These results indicate that apart from IFN-γ, all other tested cytokine concentrations are upregulated in asymptomatic malaria, as in clinical malaria. Cytokine concentrations were significantly higher in clinical malaria, whereas the IL-10 and TNF-α over parasite density ratio were higher in asymptomatic malaria.

**Figure 3:**
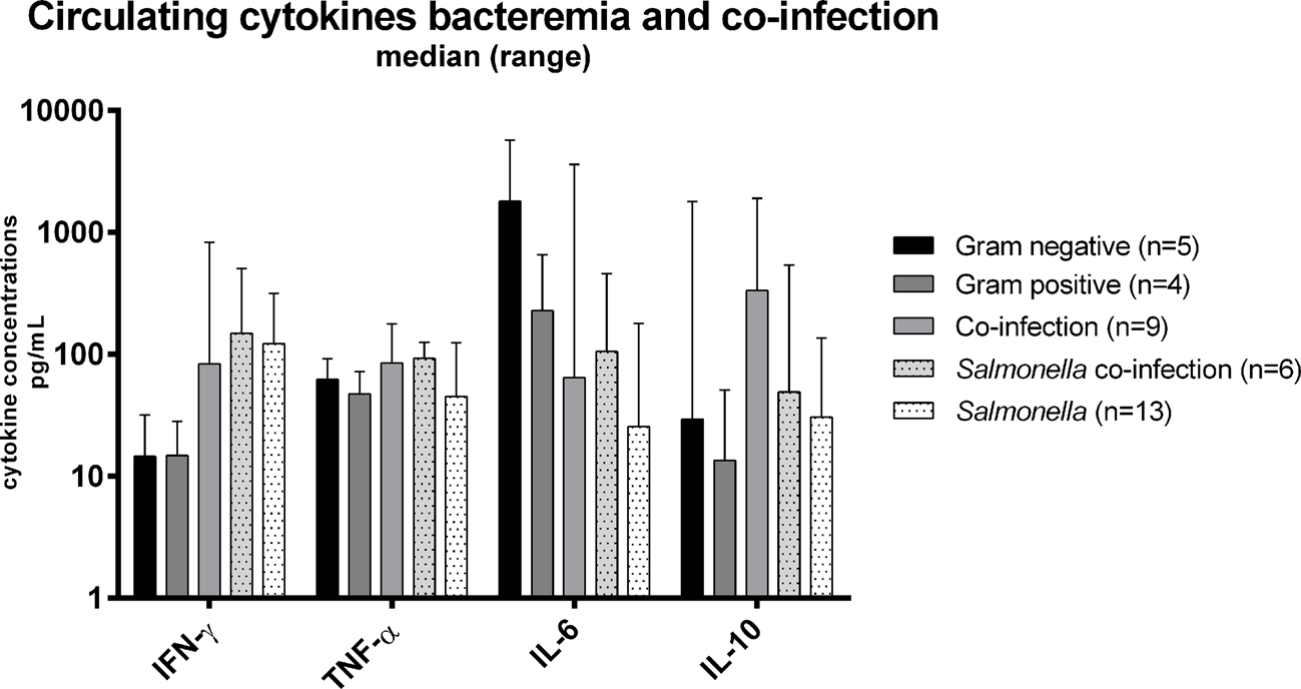
Circulating cytokine concentrations among patients below five years old with blood culture confirmed bacteremia (Gram positive, Gram negative, *Salmonella*) and malaria-bacteremia co-infection. Legend | Cytokine concentrations reported in pg/mL.

We next assessed circulating cytokine concentrations in acute febrile patients below the age of five diagnosed as malaria, bacteremia, or malarial bacteremia co-infection. Results are presented in **Table 3** and **Figure 3**. Patients with bacteremia had significantly lower IL-10 concentrations (p=0.02) compared to those with malaria parasitemia with or without bacteremia co-infection. The other circulating cytokines were not statistically different between groups.

**Table 3:** Circulating cytokines among patients with clinical malaria, bacteremia, and combined malaria-bacteremia infection.

	*(n)*	*IFN-γ*	*TNF-α*	*IL-6*	*IL-10*
Clinical malaria	114	42.9 (25.1-78.1)	71.6 (42.5-121.0)	69.2 (22.2-303.0)	439.0 (140.0-1,333.0)
Bacteremia	22	36.2 (11.3-205.0)	52.4 (27.4-71.11)	124.0 (21.3-457.0)	22.2 (8.9-100.0)
Co-infection	9	83.7 (33.6-212.0)	85.6 (67.7-107.0)	64.7 (40.2-242.0)	333.0 (85.5-707.0)
p-value		0.2	0.1	.5	<.0001
**Circulating cytokines differentiated by cause of bacteremia**					
Gram positive bacteremia	4	14.9 (9.7-25.0)	47.6 (22.7-72.4)	227.5 (124-540)	13.5 (11.9-38.6)
Gram negative bacteremia	5	14.6 (4.6-20.1)	62.6 (582-77.0)	457 (373.0-4617.0)	29.5 (5.6-174.0)
*Salmonella* bacteremia	13	45.2 (27.4-70.4)	30.6 (8.9-100.0)	14.6 (4.6-20.1)	25.6 (17.6-146.0)
*Salmonella* co-infection	6	148.4 (69.6-396.0)	92.8 (71.2-107.0)	49.2 (38.4-193.0)	104.8 (17.1-333.0)

Data is presented as median (25-75 IQR). Significance was calculated using Kruskal Wallis test. Cytokine concentrations are reported in pg/mL.

Further analysis of the different pathogens involved in bacteremia (*i.e.* Gram positive [*Streptococcus pneumoniae*, other *Streptococci*], Gram negative [*Escherichia coli, Haemophilus influenzae, Neisseria meningitidis]*, *Salmonella* spp.) showed a lower IL-6 concentration among patients with *Salmonella* bacteremia compared to the patients suffering from infections with other pathogens. Patients with *Salmonella* bacteremia also had the lowest circulating concentrations of IL-10, but the highest concentration of IFN-γ. There was no significant correlation between parasite density and cytokine levels among patients with co-infection, or *Salmonella* co-infection. These results suggest that the anti-inflammatory response (IL-10) is upregulated during clinical malaria infections while pro-inflammatory IL-6 (and TNF-α) are particularly downregulated during *Salmonella* bacteremia.

### *Ex-vivo* Cytokine Production in Participants With Asymptomatic Malaria Versus Slide Negative Controls

To explore whether circulating immune cells of asymptomatic patients with or without malaria parasitemia differed in their capacity to produce cytokines, whole blood was stimulated with various antigens, including heat killed *Staphylococcus aureus* (*S. aureus*), heat-killed *Salmonella* Typhimurium and *Escherichia coli* LPS (LPS). Samples from 180 (49.7%) participants were excluded due to contamination of the sample during the stimulation assay. The excluded participants are further detailed in **Supplementary Tables 1** and **2**: there was no significant difference in number of participants with asymptomatic malaria between included and excluded samples (n=67 *versus* n=59, respectively p=.38).

**Figure 4:**
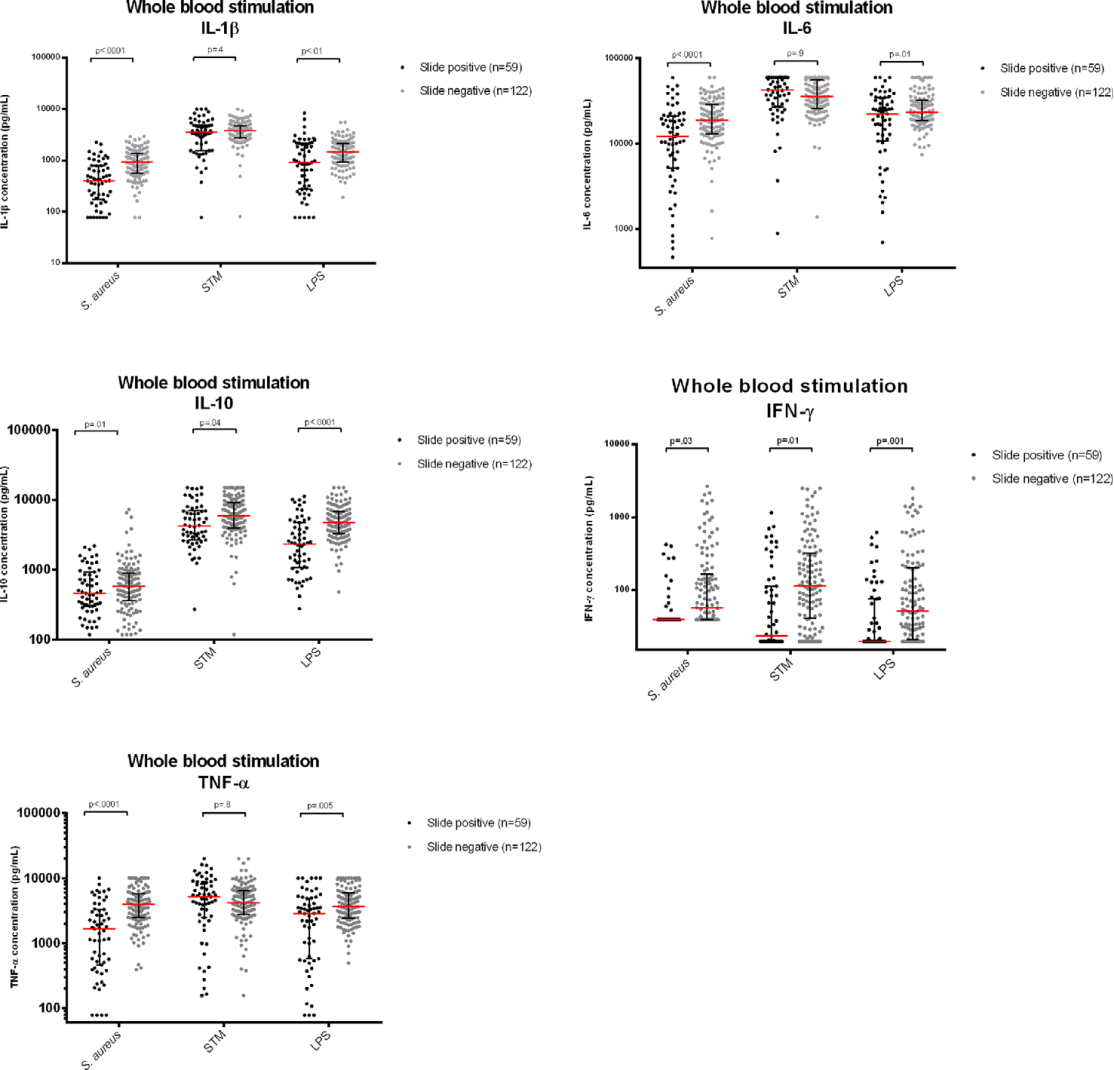
Cytokine concentrations measured in supernatants after whole blood stimulation with *Staphylococcus aureus*, *Salmonella* Typhimurium (STM) and *Escherichia coli* LPS (LPS), comparing participants with asymptomatic Malaria with thick film negative healthy controls. Legend | Cytokine concentrations reported in pg/mL. For comparison, cytokine concentrations were log-converted. P-values corrected for multiple testing using a Bonferroni test.

**Table 4** and **Figure 4** present the cytokine concentrations of asymptomatic children with or without malaria parasitemia after whole-blood stimulation with *S. aureus, Salmonella* Typhimurium and LPS, after correction for age, sex, weight, MUAC, leukocyte count, monocyte count, platelet count, hemoglobin levels and ELISA-plate layout to correct for batch effect. Height caused multicollinearity and was therefore not corrected for.

**Table 4:** Multivariable regression analyses of whole blood stimulated samples among slide negative participants and participants with asymptomatic malaria.

	Slide negative cases		Asymptomatic malaria		Coëfficiënt	95% CI	t-statistic	p-value
	*n=123*		*n=59*					
	*Staphylococcus aureus*							
	Median	25-75 IQR	Median	25-75 IQR	Coëfficiënt	95% CI	t-statistic	p-value
IFN-γ	56.3	39-157.9	39	39-39	-0.49	-0.84 - -0.15	-2.81	.03
TNF-α	3,950.4	2,460.5-5,811.3	1,710.4	505.4-3,269.0	-0.95	-1.33 - -0.56	-4.86	<.0001
IL-1β	927.3	562.3-1,362	404.1	185.4-792.1	-0.76	-1.07 - -0.44	-1.07	<.0001
IL-6	18,851.6	13,024.2-29,192.5	12,917.9	5,298.5-21,583.2	-0.65	-1.01 - -0.29	-3.57	<.0001
IL-10	575.3	353.4-887.5	432.4	299.4-849.2	-0.33	-0.60 - -0.05	-2.33	.01
	***Salmonella Typhimurium***							
	**Median**	**25-75 IQR**	**Median**	**25-75 IQR**	**Coëfficiënt**	**95% CI**	**t-statistic**	**p-value**
IFN-γ	115.1	40.8-318.7	23.3	19.5-112.9	-0.99	-1.62 - -0.37	-3.12	.01*^a^
TNF-α	4,205.7	2,767.2-6,457.8	5,332.8	2,593.4-8760.2	-0.05	-0.35 – 0.26	-0.30	.8
IL-1β	3,816.3	2,718.2-4,712.0	3,492.1	1,558.7-4,873.9	-0.09	-0.30 – 0.12	-0.86	.4*^b^
IL-6	35,579.3	25,873.2-55,766.3	42,766.3	27,196.0-60,000.0	-0.01	-0.22 – 0.20	-0.08	.9*^c^
IL-10	5,854.4	3,910.6-9,032.8	4,220.2	2,659.2-6,955.4	-0.64	-1.10 - -1.8	-2.75	.04
		**LPS**						
	**Median**	**25-75 IQR**	**Median**	**25-75 IQR**	**Coëfficiënt**	**95% CI**	**t-statistic**	**p-value**
IFN-γ	51.3	20.9-201.2	19.5	19.5-76.3	-1.10	-1.76 - -0.45	-3.32	.005*^d^
TNF-α	3,750.7	2,442.0-5,843.4	2,859.1	574.9-4,933.3	-0.67	-1.08 - -0.27	-3.28	.005
IL-1β	1,468.5	945.1-2,138.1	908.8	277.5-2,164.8	-0.53	-0.88 - 09.19	-3.08	.01
IL-6	23,390.8	18,768.9-33,449.6	23,569.1	11,374.7-34,652.3	-0.45	-0.73 - -0.17	-3.17	.01
IL-10	4,727.5	3,314.7-6,845.3	2,252.2	1,075.9-4,718.9	-0.62	-0.91 - -0.34		<.0001

*Tobit regression; *^a^41 observations censored, *^b^5 observations censored, ^* c^44 observations censored, ^* d^57 observations censored.

Each cytokine was analyzed in a separate model. All models were corrected for age, sex, weight, MUAC, leukocyte count, monocyte count, platelet count, hemoglobin levels and ELISA-plate layout to correct for batch effect. Cytokine concentrations are reported in pg/mL. For comparison, cytokine concentrations were log-converted. P-values corrected for multiple testing using a Bonferroni test.

Significantly lower cytokine responses (IL-1β, IL-6, IL-10, TNF-α and IFN-γ) to LPS and *Staphylococcus aureus* were observed among participants with asymptomatic malaria compared to controls. Participants with asymptomatic malaria had also lower IFN-γ and IL-10 responses after *Salmonella* Typhimurium stimulation, while monocyte cytokines (IL-1β, IL-6, TNF-α) were not statistically different between slide negative participants and participants with asymptomatic malaria. Likewise, we found that cytokine responses to *Salmonella* Typhimurium were generally considerably higher compared to the other two stimuli.

**Figure 5:**
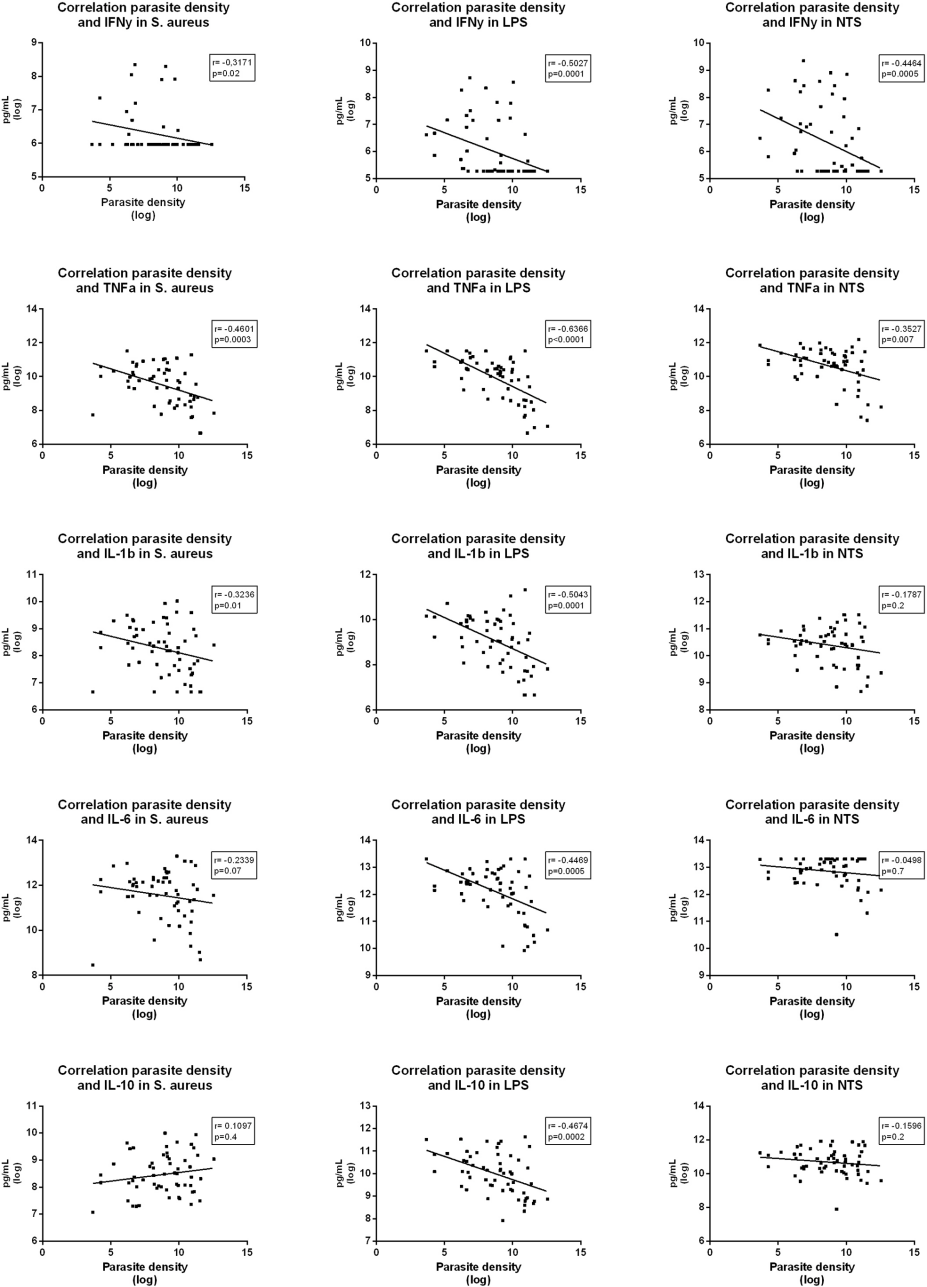
Correlations between parasite density and various cytokine levels measured in supernatants after whole blood stimulation with *Staphylococcus aureus, Salmonella* Typhimurium (STM) and *Escherichia coli* LPS (LPS) among participants with asymptomatic malaria.

In the whole-blood stimulations, parasite density was strongly negatively correlated to production of most cytokines (**Figure 5**). IL-6, IL-10 and IL-1β in samples exposed to *Salmonella* Typhimurium did not significantly correlate with parasite densities, which corresponds to the *ex-vivo* cytokine stimulation results.

These results suggest that asymptomatic malaria downregulates the cytokine production capacity of circulating immune cells after stimulation with LPS and heat killed *S. aureus*, while *Salmonella* Typhimurium downregulates the mostly lymphocyte-derived IFN-γ but had limited effect on monocyte-derived cytokines.

## Discussion

Our findings demonstrate that plasma cytokine concentrations and cytokine production capacity of circulating immune cells upon *ex-vivo* bacterial stimulation in children with asymptomatic malaria are altered compared to children without asymptomatic malaria. Further studies should be done to explore whether the observed downregulation of pro-inflammatory cytokines impairs the control of bacterial infections, in particular intracellular infections such as invasive *Salmonella*, confirming the non-benign nature of chronic malaria parasitemia (21, 22).

The study has several strengths and weaknesses. A large number of clinical cases and asymptomatic participants with or without microscopic malaria were included, allowing for comparison of different clinical phenotypes among individuals living in a highly endemic malaria area. A limitation is the number of samples that had to be excluded from *ex-vivo* cytokine production analyses due to possible contamination. The large number of contaminated cases may be explained by traces op endotoxin in heparinized collecting tubes (23) or blood sampling procedures during the field studies. The effect of excluding these samples on our data was minimal; all analyses were performed while including and excluding these isolates and results were not significantly different, but it is possible that the high number of excluded samples introduced a bias.

The study was carried out in a high malaria endemic area which is both a strength - high number of asymptomatic cases, and a weakness - submicroscopic malaria cases are likely to have been included in the “slide negative” group. The number of these sub-microscopic cases may be significant, as there is evidence of missing on average half of all *P. falciparum* infections with microscopy in endemic areas compared with PCR (24). On the other hand, excluding the submicroscopic malaria cases is likely to increase the observed differences in cytokine production capacity between asymptomatic malaria and PCR negative asymptomatic cases. A comparison with clinical cases would have been interesting, however *ex-vivo* stimulation was not done in symptomatic cases.

The introduction of seasonal malaria prophylaxis in 2016 (during our study period) may have influenced results, seeing as part of the clinical malaria samples were collected prior to the introduction of seasonal prophylaxis whereas the asymptomatic cases were collected after introduction.

The amount of blood sampled was too low to perform all analyses in duplicate. Since anemia is common among children in the study site, to sample more blood would have been unethical. However, the fact that we did not use duplicate measurements may have introduced a bias.

The *Salmonella* serotype used in the stimulation experiments was obtained from DR Congo (4) and not from the study site. However, the strain is the same invasive clade (ST-313 Lineage II) which is also most common in Burkina Faso (25) and is therefore representative for the wild types prevalent at our study site. For the detection of invasive *Salmonella* infections, blood cultures were used in the clinical cases, which only have an approximate sensitivity of 50% (26).

Several of our findings are in line with existing literature. Parasite densities among patients with clinical malaria were significantly higher compared to those with asymptomatic malaria. Likewise, patients with clinical malaria infection had significantly higher circulating cytokine concentrations compared to participants with asymptomatic malaria. We found a significant positive relation between parasite densities and circulating cytokine levels (IL-10), both in asymptomatic (27) and clinical malaria cases (28, 29). Patients with combined malaria-bacteremia generally had the highest circulating cytokine concentrations compared to patients with malaria or bacteremia alone. Among these patients, cytokine concentrations did not statistically correlate with parasite density, juxtaposed to all other analyses in which cytokine concentrations either positively or negatively correlated to parasite density. Together, these results suggest that once co-infection is established, bacteremia is the primary determinant of cytokine concentrations, and the downregulatory effect of malaria is no longer clinically relevant.

Comparing circulating cytokine concentrations among asymptomatic participants, we also found significantly higher pro- and anti-inflammatory cytokine concentrations in malaria microscopy positive participants compared to microscopy negative controls (30). The largest difference was found for IL-10, confirming the presence and importance of an anti-inflammatory response in asymptomatic malaria to dampen the pro-inflammatory response and prevent the development of clinical disease (31, 32). The cytokine/parasite density ratios also pointed in this direction as the IL-10/parasite density ratio was highest in asymptomatic malaria cases while the pro-inflammatory IL-6/parasite density ratio was highest in patients with clinical malaria. Previous studies demonstrate that high levels of IL-6 as well as TNF-α contribute to complications of malaria such as cerebral malaria (20).

Cytokine concentrations in the supernatant of stimulated whole blood of asymptomatic participants indicate a downregulation of cytokine production capacity of immune cells in asymptomatic participants with positive malaria microscopy compared to those with negative microscopy, consistent with the hypothesis that *Plasmodium* induces immune hyporesponsivity to bacterial antigenic stimulation. Metenou et al (33 *)* studying filarial infected patients with and without asymptomatic malaria also found evidence using flow cytometry of a lower cytokine production capacity of immune cells in case of concurrent malaria parasitemia. Other previous research focused on clinical malaria, demonstrating that circulating blood samples of clinical malaria patients produced a lower cytokine response (34–36) and contained less TNF-α and IL-6 producing monocytes compared to healthy controls upon stimulation with *E. coli* LPS (37). These cytokines play a crucial role in host defense and an impaired cytokine production capacity may therefore contribute to the clinically observed increased susceptibility to bBSI in asymptomatic malaria (34, 37), an effect we found to be inversely related to parasite density. While the current study also found a downregulation in cytokine responses, it is difficult to interpret how this would affect clinical outcome without a clinical study. On one hand a downregulation of the early pro-inflammatory cytokines could impair the host response leading to an increased susceptibility to bacterial infections. Alternatively, the lower cytokine concentrations could be sufficient to contain bacterial infection but prevent further damage due to septicemia.

Specific cytokines are known to play a crucial role in the host defense against intracellular pathogens like *Salmonella.* The early immune response to *Salmonella* is driven by TNF-α, with the purpose to contain a localized infection (9, 37). IFN-γ, produced by antigen-specific helper T cells, activates macrophages thereby inducing intracellular killing mechanisms against *Salmonella* (38–40). Simultaneously, *Salmonella* upregulates the IL-10 production of monocytes, compromising the production of reactive oxygen radicals and intracellular killing capacity of monocytes (41, 42). The relatively low level of IL-6 among patients with iNTS as observed in this study may partially explain why patients with iNTS often present with relatively mild symptoms, as IL-6, together with IL-1 are considered the major endogenous pyrogens (43, 44).

Numerous studies explored the effect of malaria on immune cells, among others the effect of the malaria pigment hemozoin (45). Hemozoin causes release of peroxidation derivatives in mononuclear cells thereby impairing various functions of these cells (46). Hemozoin is detected in mononuclear cells of clinical and asymptomatic malaria cases reflecting the level of parasitemia (47). Mononuclear cells are of special importance to *Salmonella* as it invades and survives within these cells. It is therefore remarkable that that stimulation with *Salmonella* Typhimurium among participants with asymptomatic malaria resulted in less IFN-γ and IL-10 production compared to LPS and *S. aureus*, while no significant difference was observed among TNF-α, IL-1β and IL-6 production capacity. The latter cytokines are primarily produced by monocyte/macrophages while T cells mostly produce IFN-γ. It must be noted that we did not assess which cells were responsible for cytokine production in the *ex-vivo* experiments and we can therefore not rule out that T-cells may have contributed to the cytokine concentrations in supernatants. Our findings suggest that the downregulation of the IFN-γ axis contributes to the increased risk for iNTS among patients with asymptomatic malaria. The importance of the IFN-γ axis was also observed in other studies assessing genetic risk profile for iNTS (48). A previous study (37) suggests an important role for TNF-α as a risk factor for iNTS in malaria. That study was however done using *E. coli* LPS instead of heat-killed *Salmonella*, which may account for the difference in results.

## Clinical Implications

Asymptomatic malaria is common in sub-Saharan Africa, with a reported prevalence up to 75% (13). Asymptomatic malaria has long been associated with immunity against malaria while its possible negative consequences are only more recently recognized (21, 22). The observed increase in circulating cytokine concentrations among patients with asymptomatic malaria supports the presence of a pro-inflammatory state with possible consequences on erythropoiesis and decreased hemoglobin levels. Our present data demonstrate that asymptomatic malaria modulates cytokine production capacity of immune cells in response to stimulation with bacterial pathogens that are common in SSA, which may influence the early immune response to bacterial pathogens. A next step in proving this theory may be to compare *ex-vivo* cytokine concentrations among patients with asymptomatic malaria before and after treatment for malaria. Conversely, the presence of bacteremia may also affect asymptomatic malaria infections: our findings of relatively low circulating levels of IL-10 and IFN-γ in patients with bacteremia indicate a compromised host defense against malaria and thus a less restricted malaria infection. Treatment of asymptomatic malaria may therefore not only reduce the malaria reservoir and malaria transmission, but also protect against the effects of malaria parasitemia on the immune system.

## Data Availability Statement

The original contributions presented in the study are included in the article/**Supplementary Material**. Further inquiries can be directed to the corresponding authors.

## Ethics Statement

Both study protocols were approved by the National Health Ethics Committee of Burkina Faso (ref 2016-01-006 and 2015-01-006, respectively). The diagnostic accuracy study was furthermore approved by the Internal Review Board of IRSS (ref A03-2016/CEIRES), the Ethical Committee of the Antwerp University Hospital (ref 15/47/492), and the Institutional Review Board of the Institute of Tropical Medicine Antwerp (ref 1029/15). Written informed consent to participate in this study was provided by the participants’ legal guardian/next of kin.

## Author Contributions

BK, AP, MB, QM, and AV were responsible for study design and execution of the cross-sectional study. AP, BK, SD, HT, JJ, QM, and AV were involved in study design of the diagnostic accuracy study. BK, SD, and OT were responsible for study management in Burkina Faso. RA, MN, and LJ were involved in preparation of laboratory techniques and quality control. Analyses and writing were done by AP, BK, MB, QM, and AV. All authors contributed to the article and approved the submitted version.

## Funding

AV and QM have a non-restricted research grant from SYSMEX, which funded the current study. Both the diagnostic accuracy study and the cross-sectional study were funded by a non-restricted research grant from SYSMEX company.

## Conflict of Interest

The authors declare that the research was conducted in the absence of any commercial or financial relationships that could be construed as a potential conflict of interest.

## Supplementary Material

Supplementary Table 1 Baseline characteristics of participants with cytokine readout on non-stimulated samples.

Supplementary Table 2 Multivariable regression analyses of whole blood stimulated samples among slide negative participants and participants with asymptomatic malaria, after correction for monocyte population and plate lay-out (including all cases, also the ones with possible contamination).
