# Supplementary material for: Altered Ex-Vivo Cytokine Responses in Children With Asymptomatic Plasmodium falciparum Infection in Burkina Faso: An Additional Argument to Treat Asymptomatic Malaria?
Source: Front Immunol. 2021 Jun 9;12:614817. doi: 10.3389/fimmu.2021.614817 (PMC8220162; doi:10.3389/fimmu.2021.614817)
Supplement: Supplementary Table 1 — Baseline characteristics of participants with cytokine readout on non-stimulated samples. [file Table_1.docx]

**Supplementary Table 1. Baseline characteristics of participants with cytokine readout on non-stimulated samples.** Data is presented as mean/SD in case of normally distributed data and median (25-75 IQR) in case of not normally distributed data. Significance value calculated using chi-2 for binary values, Mann-Whitney U-test in case of not normally distributed and student’s t-test in case of normally distributed values.

|  | Non contaminated samples | Contaminated  samples | p-value |
| --- | --- | --- | --- |
|  | ***n=181*** | ***n=180*** |  |
| Male* | 90 (49.7%) | 89 (49.4%) | .91 |
| Age (months) | 25.7 (22.7-39.9) | 25.3 (21.7-41.2) | .8 |
| Weight | 11.7 (10.0-12.9) | 11.0 (9.3-12.5) | .**009** |
| Height | 83 (78-91) | 83 (78-91) | .8 |
| MUAC | 15.5 (1.2) | 15.0 (1.1) | ***.01*** |
| Axillary temperature | 36.7 (36.3-37.1) | 36.3 (36.0-36.8) | ***<.0001*** |
|  |  |  |  |
| Asymptomatic malaria | 59 (32.4%) | 67 (37.2%) | .34 |
| Parasite density | 8879 (997-34,637) | 1432 (485-7,682) | ***.0004*** |
|  |  |  |  |
| WBC count | 9.7 (2.7) | 10.4 (3.5) | .1 |
| Monocyte count | 0.9 (0.4) | 1.0 (0.4) | .3 |
| Neutrophil count | 2.8 (1.6) | 2.8 (1.9) | .8 |
|  |  |  |  |
| Baseline circulating cytokines | | | |
| IL-6 | 1.7 (0.9-3.7) | 1.5 (0.8-2.7) | **.01** |
| IL-10 | 7.3 (3.1-30.6) | 4.8 (1.7-23.4) | **<.0001** |
| TNF-alpha | 28.5 (21.5-38.8) | 26.8 (19.8-36.6) | ***.04*** |
| IFN-gamma | 2.1 (2.1-2.4) | 2.1 (2.1-2.1) | .3 |
|  |  |  |  |

* calculated using chi2

MUAC: Mid-upper arm circumference | WBC: White blood cells
