# Supplementary material for: Altered Ex-Vivo Cytokine Responses in Children With Asymptomatic Plasmodium falciparum Infection in Burkina Faso: An Additional Argument to Treat Asymptomatic Malaria?
Source: Front Immunol. 2021 Jun 9;12:614817. doi: 10.3389/fimmu.2021.614817 (PMC8220162; doi:10.3389/fimmu.2021.614817)
Supplement: Supplementary Table 2 — Multivariable regression analyses of whole blood stimulated samples among slide negative participants and participants with asymptomatic malaria, after correction for monocyte population and plate lay-out (including all cases, also the ones with possible contamination). [file Table_2.docx]

**Supplementary Table 2.** Multivariable regression analyses of whole blood stimulated samples among slide negative participants and participants with asymptomatic malaria, after correction for monocyte population and plate lay-out (including all cases, also the ones with possible contamination)

|  | Slide negative cases | | | Asymptomatic malaria | | | | p-value |
| --- | --- | --- | --- | --- | --- | --- | --- | --- |
|  | n=236 | | | n=126 | | |  |  |
|  | ***Staphylococcus*** ***aureus*** | | | | | | |  |
|  | **Median** | **25-75 IQR** | | **Median** | **25-75 IQR** | | |  |
| IFNy | 49.2 | 39.0-131.1 | | 39 | 39-59.3 | | | <.0001 |
| TNFa | 4,624.9 | 2,946.0-7,188.0 | | 3,105.9 | 1,553.6-6,147.6 | | | <.0001 |
| IL-1b | 1,600.9 | 852.4-2,787.1 | | 1,173 | 404.1-2,598.5 | | | <.0001 |
| IL-6 | 26,619.8 | 16,165.0-39,989.2 | | 28,765.9 | 12,161.5-47,826.4 | | | .02 |
| IL-10 | 1,324.4 | 505.5-4800.4 | | 1,357 | 430.5-4,039.9 | | | .02 |
|  | ***Salmonella Typhimurium*** | | | | | | |  |
|  | **Median** | | **25-75 IQR** | **Median** | | **25-75 IQR** | |  |
| IFNy | 82.1 | | 24.4-226.7 | 37.3 | | 19.5-138.7 | | .001 |
| TNFa | 3,652.4 | | 2,201.6-5,672.8 | 4,461.7 | | 2,167.7-7,583.3 | | .3 |
| IL-1b | 3,734.2 | | 2,701.4-4,887.4 | 3,874.2 | | 2,721.5-5,738.7 | | .8 |
| IL-6 | 31,216.3 | | 23,715.7-45,949.6 | 41,366.9 | | 27,901.1-60,000.0 | | .1 |
| IL-10 | 5,864.1 | | 3,966.1-8,929.7 | 5,425.3 | | 3,051.9-8,158.2 | | .001 |
|  |  | | **LPS** | | |  | |  |
|  | **Median** | | **25-75 IQR** | **Median** | | **25-75 IQR** | |  |
| IFNy | 42.4 | | 19.5-117.5 | 22.4 | | 19.5-79.2 | | .01 |
| TNFa | 3,524.5 | | 2,281.1-5,640.3 | 3,156.6 | | 1,387.8-5,118.6 | | .008 |
| IL-1b | 1,755.0 | | 1,122.8-2,643.6 | 1,677.8 | | 835.6-2,598.8 | | .05 |
| IL-6 | 22,747.5 | | 17,882.9-31,914.7 | 24,761.9 | | 18.350.4-37,922.4 | | .04 |
| IL-10 | 4,822.6 | | 3,353.8-7,381.2 | 3,605.6 | | 1,798.9-6,031.2 | | <.0001 |
